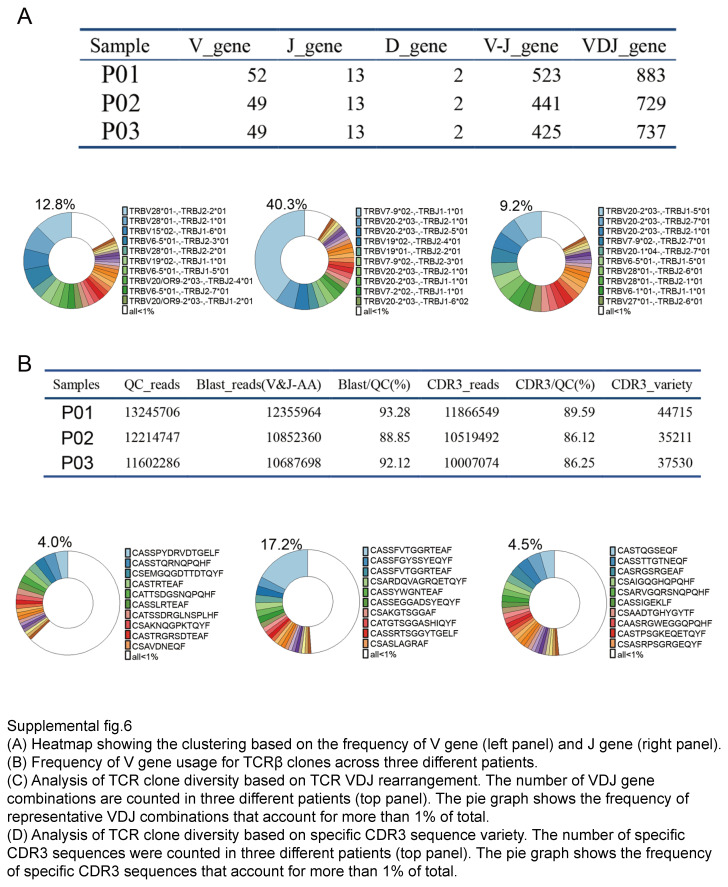# Single-cell map of diverse immune phenotypes in the metastatic brain tumor microenvironment of nonsmall-cell lung cancer

**DOI:** 10.1097/JS9.0000000000002088

**Published:** 2024-09-23

**Authors:** Liang Wang, Min Chao, Run-Run Han, Lei Li, Lei Dong, Fan Chen, Ming-Zhu Jin, Li Gao, Yuan Wang, Da-Yun Feng, Gang Zhu, Wei Guo, Wen-Jian Zhao, Shi-Jia Jin, Dong-Ping Wei, Wei Sun, Jin-Xiang Dai, Wei-Lin Jin

**Affiliations:** a Department of Neurosurgery, Tangdu Hospital, Fourth Military Medical University, Xi’an, People’s Republic of China; b Frontier Medical Innovation Center, Tangdu Hospital, Fourth Military Medical University, Xi’an, People’s Republic of China; c Division of Stem Cell Biology, Institute for Genetic Medicine, Hokkaido University, Sapporo, Hokkaido, Japan; d School of Public Health, Health Science Center of Xi’an Jiaotong University, Xi’an, People’s Republic of China; e Key Laboratory of Trace Elements and Endemic Diseases of National Health Commission and Collaborative Innovation Center of Endemic Diseases and Health Promotion in Silk Road Region, Xi’an, People’s Republic of China; f Department of Pathology, Ruijin Hospital, Shanghai Jiao Tong University School of Medicine, Shanghai, People’s Republic of China; g Department of Obstetrics and Gynecology, Renji Hospital, School of Medicine, Shanghai Jiao Tong University, Shanghai, People’s Republic of China; h Shanghai Jiao Tong University School of Medicine, Shanghai, People’s Republic of China; i Medical Research Center, The First Affiliated Hospital of Wenzhou Medical University, Wenzhou, Zhejiang Province, People’s Republic of China; j Department of Neurosurgery, Shanghai Institute of Neurosurgery, Changzheng Hospital, Second Military Medical University, Shanghai, People’s Republic of China; k Human Biology Division, Laboratory for the Study of Metastatic Microenvironments, Fred Hutchinson Cancer Research Center, Seattle, USA; l Institute of Cancer Neuroscience, Medical Frontier Innovation Research Center, The First Hospital of Lanzhou University, The First Clinical Medical College of Lanzhou University, Lanzhou, People’s Republic of China

## Introduction

Highlights

- This is the first time that the immune status of brain metastases from LUAD and breast cancer has been studied through large-scale parallel sequences.
- We found that tumor stromal cells in lung adenocarcinoma brain metastases have unique transcriptomic characteristics, which distinguish them from primary tumors.
- The expansion of immunosuppressive macrophages and the lack of activation of T cells may explain the immune escape shown by brain metastasis.

Nonsmall-cell lung cancer (NSCLC), especially adenocarcinoma (LUAD), contributes to almost half of the metastatic brain tumors diagnosed^1^, which patients always accompanied with poor prognosis. While small molecule kinase or immune checkpoint inhibitor, which play important therapeutic roles in NSCLC, have eventually developed drug resistance in NSCLC patients, especially brain metastases (BMs) patients^2^. The differential immune microenvironment (IME) between BMs and primary NSCLC may be the main factor contributing to treatment resistance in metastatic lesions^3,4^. This study aims to analyze the different of IME in BMs with NSCLC, and we found that CNS metastasis patients exhibited a lack of T cell infiltration and activation, and a unique signature of TAMs in the BMs from LUAD.

## Results

### Immune landscape in BMs of lung adenocarcinoma

**Figure 1:**
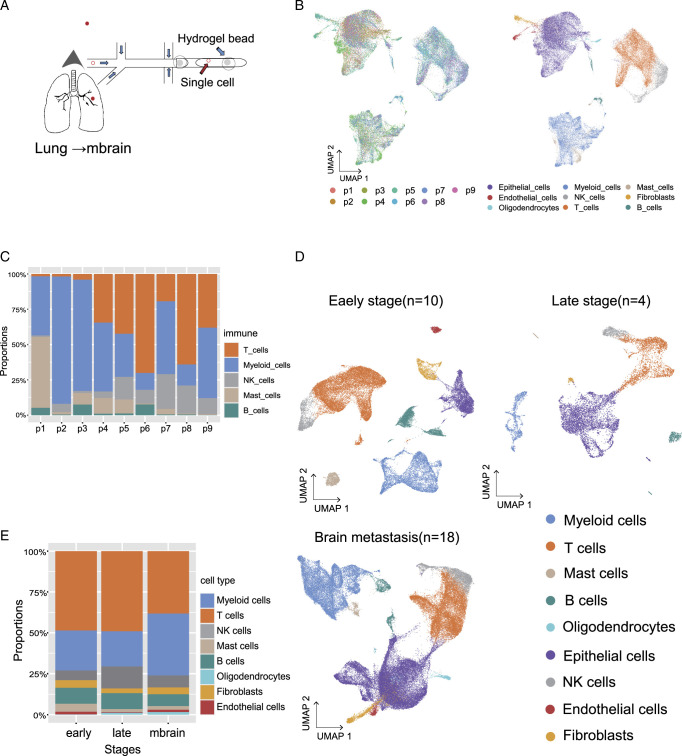
Immune landscape of brain metastasis of lung adenocarcinoma. (A) Tissue samples were taken from the brain tissue (temporal lobe/parietal lobe/occipital lobe) and transferred from lung tissue to the back of the brain in patients with lung adenocarcinoma. (B) Uniform manifold approximation and projection (UMAP) of the 51 787 cells profiled here, with nine color codes corresponding to nine patients with BMs of LUAD (B left panel: P1–P9); different color codes correspond to different cell types (B right panel). (C) (before integration) Proportions of major cell types for BMs of LUAD patient’s tumor infiltrating immune cells, colored by cell type. (D) UMAP of 37 501 cells from early-stage LUAD (ELC) and 10 699 cells from later-stage LUAD (LLC), along with 73 691 cells from brain lesions of LUAD (LBM). (E) Proportions of major cell types for each cluster derived from (D) as combined all tumor-infiltrating immune cells from all three different stages of LUADs, colored by cell type

Samples were collected from surgical resection with BMs of LUAD (*n*=9). A total of 92 310 cells were subjected to scRNAseq using the 10× Genomics in Drop platform, and 51 787 cells were used to construct the map (Fig. 1A and Supplementary Methods, Supplemental Digital Content 1, http://links.lww.com/JS9/D454). In addition to malignant cells (16 938 cells), immune cells [myeloid, T cells, B cells, Mast cells, and Nature killer cells (NK)], oligodendrocytes, epithelial cells, endothelial cells, and fibroblasts were identified (Fig. 1B). The tumor-resident immune cells (29 825 cells) included myeloid cells (20.73%), T cells (24.80%), and B cells (1.53%) (Fig. 1C and Table S1, Supplemental Digital Content 2, http://links.lww.com/JS9/D455). As we can see, the composition distribution of immune cells was varied in different patients, while myeloid cells accounted for the majority of immune cells, which was consisted with the bulk RNA analysis of the BMs with LUAD (*n*=6) (Fig. S2C, Supplemental Digital Content 3, http://links.lww.com/JS9/D456 and S3, Supplemental Digital Content 4, http://links.lww.com/JS9/D457).

### Immune microenvironment of BMs is unique compared to that of the primary LUAD

It is known to that lack of T lymphocyte and expansion of TAMs is the main feature in the BMs of LUAD^4,5^. IHC assays of the nine BMs from LUAD and a tissue array containing 35 primary NSCLC samples, were used to display that T cells (CD3^+^), B cells (CD20^+^), and macrophages (CD68^+^) in BMs were significantly decreased, compared with primary LUAD (Table S2, Supplemental Digital Content 5, http://links.lww.com/JS9/D458, Fig. S3, Supplemental Digital Content 4, http://links.lww.com/JS9/D457, and Supplementary Methods, Supplemental Digital Content 1, http://links.lww.com/JS9/D454). Then we reanalyzed published scRNAseq datasets from early and late-stage LUAD^6^, along with BMs (integrate published^6^ and our own data) (Fig. 1D). The cluster distribution displayed that T and B lymphocytes presented a higher frequency in the TME in both primary LUAD (58.34%, 58.83% vs. 45.50%), while the proportion of myeloid cells were increased in BMs (37.79% vs. 24.41%, 21.57%) (Fig. 1E and Table S3, Supplemental Digital Content 6, http://links.lww.com/JS9/D459).

### Immune cell mapping reveals a distinct macrophage/microglia signature in BMs of LUAD

**Figure 2:**
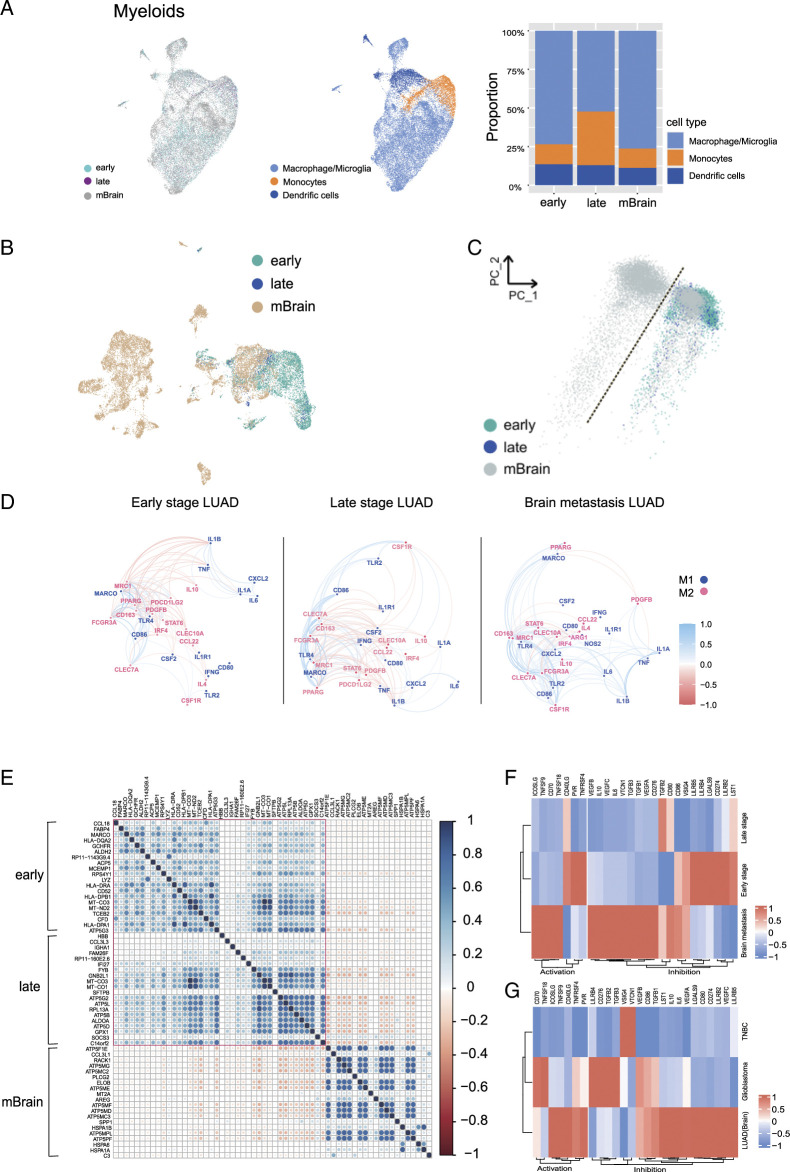
Molecular profile of the myeloids cells and TAMs in different stages of lung adenocarcinoma. (A) umap and features plots showing 12 687 myeloid cells from different clusters of LUADs (early, late, and mBrain). Color-coded by their associated clusters (left) or the expression of marker genes for some cell types (right). (B) UMAP plot showing myeloids cells from different stages of LUAD (early, late, and mBrain). UMAP plot showing myloids cells from different cell types of LUADs (middle); Proportions of cell types for each cluster from all three different stages of LUADs, colored by cell type (right). (C) UMAP and principle component analysis (PCA) based on transcriptomes of TAM cells from different stages of LUAD patients, colored by stages. (D) Differential gene expression correlation analysis reveals unique TAM signature in three different stages of LUAD. (E) Heatmap showing DEGs in each cluster of TAMs cellsfromdifferent stages of LUAD (ELC, LLC, and LBM). (F, G) Heatmap showing expression of distinct T cell costimulatory and coinhibitory ligands on macrophages from brain metastases of LUAD (LBM) versus primary lung cancers (ELC and LLC) (left), and also from brain lesions from LUAD (LBM), breast cancer brain metastases (TNBC), and glioma.

It is urgent to clarify the molecular composition of the myeloid cells that contributed to the process of LUAD to BMs. With 25 946 myeloid cells revealed that macrophage/microglia (CD68, CD163, and APOE) represented the most prevalent cluster in metastatic brain lesions, which were extracted to perform TAM analysis again (Fig. 2A, B). The TAM clusters from primary LUAD were separated from BMs by PCA analysis (Fig. 2C). The further analysis showed that ‘alternatively activated’ (M2) macrophages (CD163, CSF1R, and CD276^7^) were increased in BMs, where ‘traditional activated’ (M1) macrophages (CCL3/CCL4) were enriched in primary LUAD (Fig. 2D and Table S4, Supplemental Digital Content 7, http://links.lww.com/JS9/D460).

Additionally, in contrast to the primary LUAD, the macrophages in BMs highly expressed mitochondrial ATP synthase subunit (ATP5F1E; ATP5MG), and ATP biosynthetic process (Fig. 2E, Fig. S4C, Supplemental Digital Content 8, http://links.lww.com/JS9/D461, and Table S5, Supplemental Digital Content 9, http://links.lww.com/JS9/D462). While in primary LUAD, biological process of antigen presentation was upregulated (Fig. S4A, B, Supplemental Digital Content 8, http://links.lww.com/JS9/D461). Our analysis reveals the unique polarization states of macrophages in BMs of LUAD.

### Molecular profiles of the macrophages at different LUAD stages reveal brain-specific TAMs lack conventional T cell costimulatory molecules

To find the causes of decreased T cells in the metastatic brain lesions, we further analyzed bulk RNAseq data from 14 early and 11 late-stage primary LUAD from the TCGA database, and 6 BMs from LUAD patients. The analysis showed that NF-κB signaling, and MHC I were decreased in BMs, where purine ribonucleotide synthesis was upregulated (Fig. S5C, D, Supplemental Digital Content 10, http://links.lww.com/JS9/D463). The results illustrated that the lack of immune costimulatory factors in the myeloid cells should be account for the decreased lymphocyte in BMs, which was also confirmed in BMs from triple-negative breast cancer (TNBC) (Fig. 2G). We further found that macrophage in both early-stage LUAD and brain lesions were lack of ligands for T cell stimulatory, which were responsible for the development of exhausted T cells (Fig. 2F and Table S5, Supplemental Digital Content 9, http://links.lww.com/JS9/D462). These results disclosed the noninflamed feature of BMs from LUAD. The macrophage in metastatic TME also lacks expression of T cell costimulatory factors.

### Unique immune cell profiles in BMs as compared to glioblastomas

**Figure 3:**
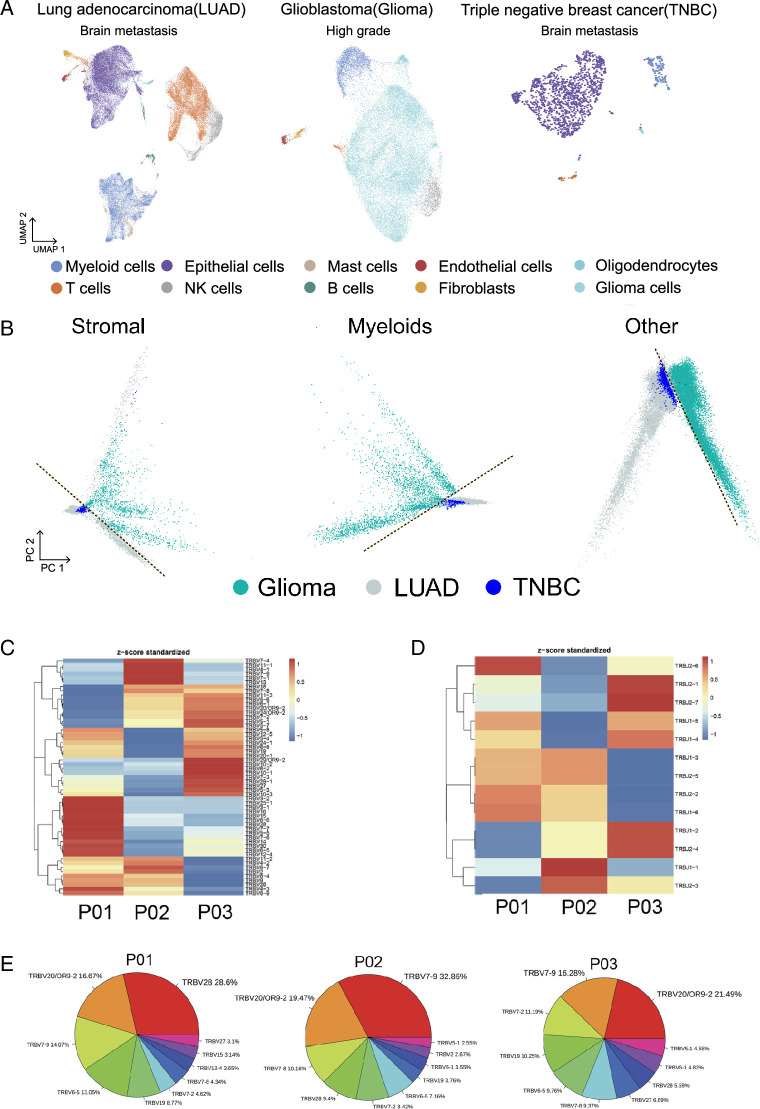
Unique immune cell profile in BMs comparing with GBM, and B cell clonality analysis of BMs from LUAD. (A) UMAP showing clusters of single cells from resected lesions of LUAD brain metastases (9× patient, 51 787cells), GBM (9× patients, 24 725 cells), and TNBC brain metastases (1× patient, 1569 cells). (B) PCA analysis of stromal cells, myeloids, and other cells from the brain metastases of Glioma, LUAD, and TNBC. UMAP showing clusters and cell types of all cells from three types of brain tumors (LUAD, Glioma, and TNBC). (C, D) Heatmap showing the clustering based on the frequency of V gene (C) and J gene. (E) Frequency of V gene usage for BCR clones across three different patients.

We found both BMs from LUAD and TNBC sharing the expression of T cell inhibitory factors but not the glioblastoma (GBM). To compare the molecular profiling, caused for TME reshaped of BMs, we took a scRNAseq of resected brain lesions of TNBC patients (Fig. S1, Supplemental Digital Content 11, http://links.lww.com/JS9/D464), and nine published GBM scRNAseq datasets^8,9^. Consistent with our findings in BMs from LUAD, we also found that there were only a tiny number of T and B lymphocytes, however, myeloid cells were abundant (Fig. 3A). The stromal cells and myeloid cells in the BMs from LUAD and TNBC were transcriptionally closer to each other than to GBM despite the different origins (Fig. 3B). Collectively, the IME of BMs is shaped by the intrinsic properties of the tumor, which are inherent from its origins, rather than the local microenvironment.

### B and T cells undergo clonal expansion in brain metastases of LUAD patients

T‐cell receptors (TCR) and B‐cell receptors (BCR) are the key molecules responsible for clonal expansion, affinity maturation and activation in adaptive immunity^10^. To understand the clonal expansion of T and B cells in the brain lesions of LUAD, V(D)J rearrangements within the BCR/TCRβ locus were amplified with V-gene-specific and J-gene-specific primers. Among the three BMs from LUAD, a total of 47 702 unique clonotypes were detected (Fig. 2C, D), and the clonality (BCR: 0.57; TCR: 0.43) was generally higher than primary LUAD (BCR: 0.07; TCR: 0.3)^10^. The heterogeneity of BCR genes across the three patients was also evident in the clustering of V or J gene, the diverse frequency of V genes, and the rearrangements of VDJ (Fig. 2E and Fig. S6A, Supplemental Digital Content 12, http://links.lww.com/JS9/D465). Despite the tiny number of T and B cells in BMs of LUAD, clonal expansion of T and B cells is apparent across the three patients analyzed by scRNAseq.

## Conclusion

In summary, we constructed the first tumor microenvironment landscape of BMs of LUAD and TNBC by massive parallel scRNAseq. We revealed that BMs associated macrophages expressed unique sets of immunosuppressive ligands (CD276 and VTCN1), comparing with primary tumors (PDL1, CD80, and LILRB2/5). Despite the lack of T and B cells in BMs of LUAD and TNBC, abundant TCR and BCR repertoire were identified. The expansion of immune suppressive macrophage and lack of T cell activation may explain the immune escape of the BMs. Our study also identified some potential targets for immunotherapies against BMs. A comprehensive understanding of the composition and evolution of IME in brain metastases will guide the design of new therapeutic strategies to target these deadly diseases.

## Ethical approval

This study was carried out in accordance with The Code of Ethics of the World Medical Association (Declaration of Helsinki) and was approved by the ethics committee of our Hospital (No. TDLL-201902-08).

## Consent

Not applicable.

## Source of funding

This study was supported by the High-Level Talent Introduction Funds from the First Hospital of Lanzhou University (Wei-Lin Jin), the National Natural Science Foundation of China (No. 81772661 to Liang Wang, No. 81602193 to Wei Sun, and No. 81872393 to Dong-Ping Wei), the Science Fund for Distinguished Young Scholars of Shaanxi Province (No. 2023-JC-JQ-68 to Liang Wang), Youth Innovation Team of Shaanxi Provincial Higher Education Institution (2022-61to Liang Wang). Jin-Xiang Dai is supported by a Postdoctoral Breakthrough Award by the DoD’s BCRP (W81XWH-18-1-0028).

## Author contribution

R.R.H., L.L., L.D., D.Y.F., and M.Z.J.: prepared the sample and performed in house characterization; G.Z., W.G., Y.W., M.C., L.G., S.J.J., D.P.W., and W.S.: performed synchrotron experiments with the assistance of E.O. and Ph.S.; G.S., L.P., and G.C.: performed and discussed the experiments; F.C.: drafted the manuscript; L.W., J.X.D., and W.L.J.: supervised the work. All authors have contributed and approved the final version of the manuscript.

## Conflicts of interest disclosure

The authors declare no conflicts of interest.

## Research registration unique identifying number (UIN)

1. Name of the registry: not applicable.

2. Unique identifying number or registration ID: not applicable.

3. Hyperlink to your specific registration (must be publicly accessible and will be checked): not applicable.

## Guarantor

Liang Wang.

## Data availability statement

The single-cell RNA-seq data and bulk RNA-seq data used in this study have been deposited in the Gene Expression Omnibus (GEO) under the accession number GSE143423 (only three cases were uploaded) and GSE141685, respectively. Raw image files used in the figures that support the findings of this study are available from the corresponding authors upon reasonable request.

## Provenance and peer review

Not commissioned.

## Supplementary Material

**Figure:**
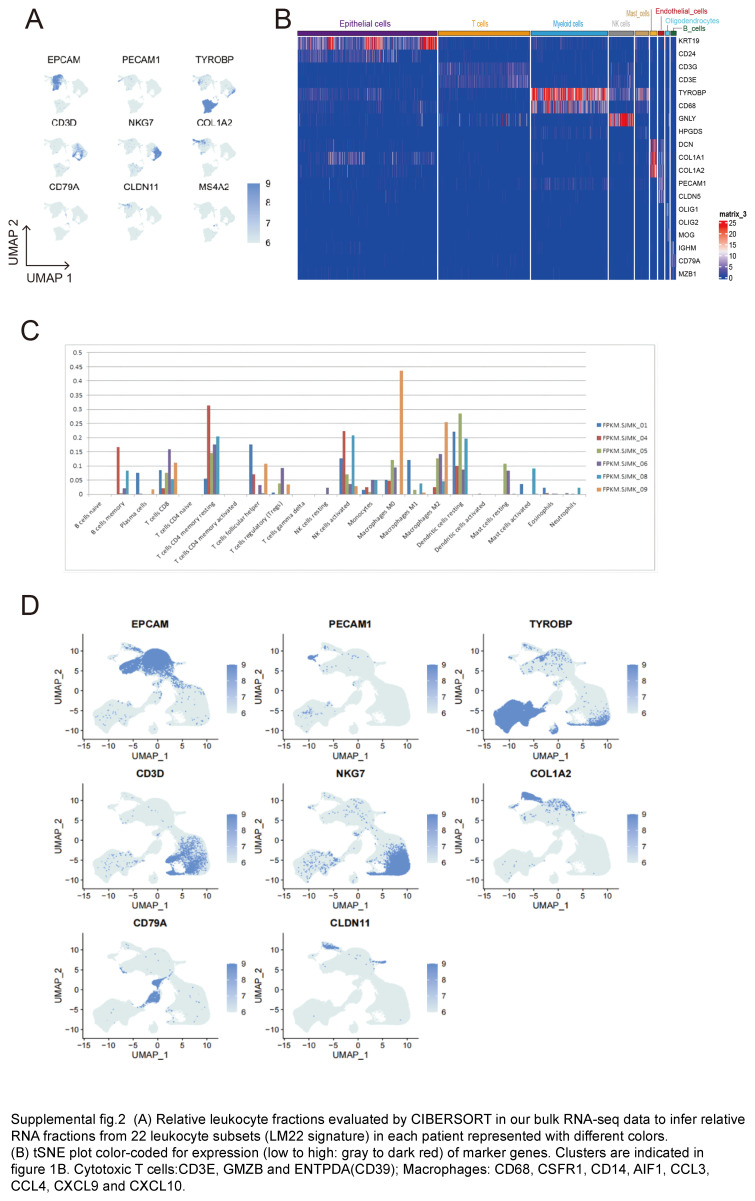


**Figure:**
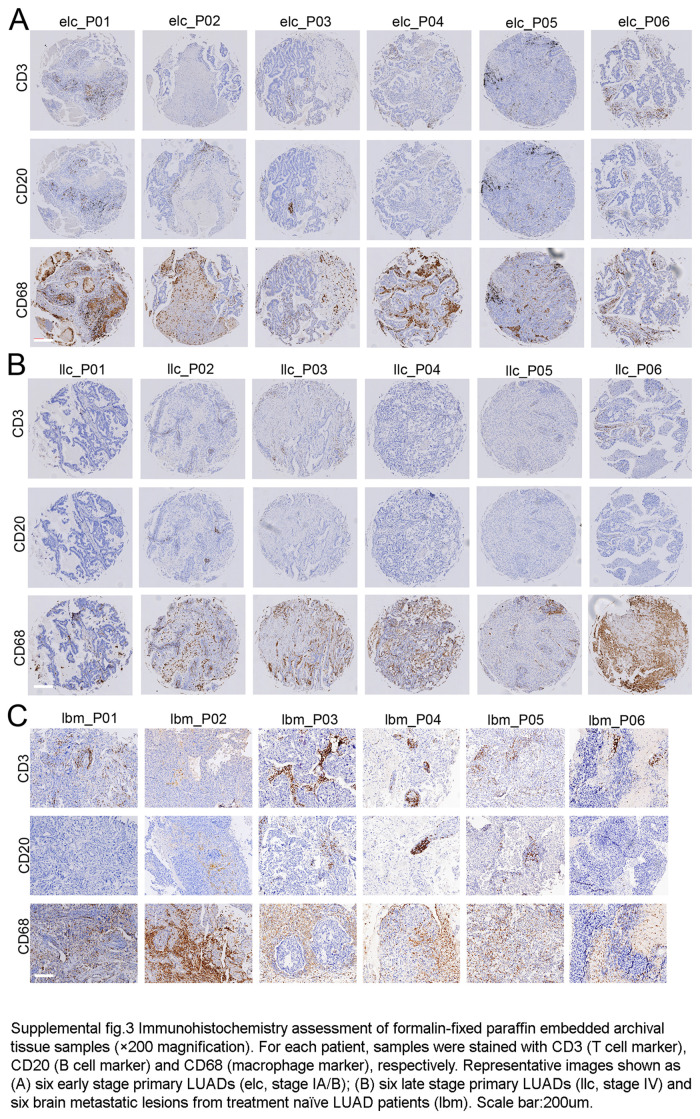


**Figure:**
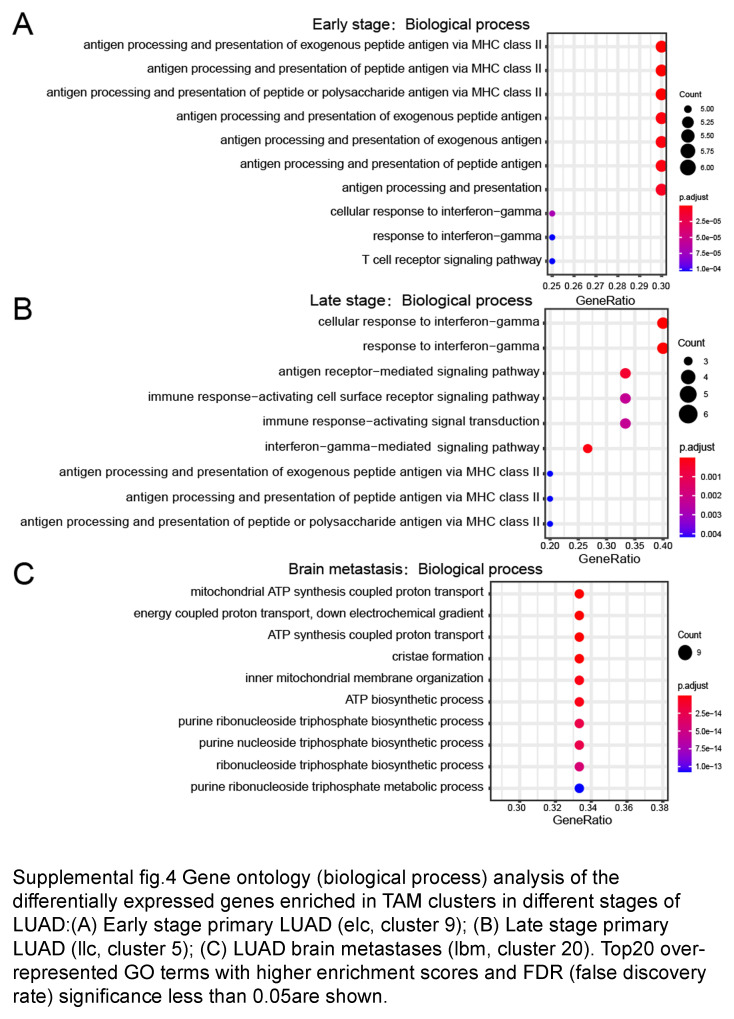


**Figure:**
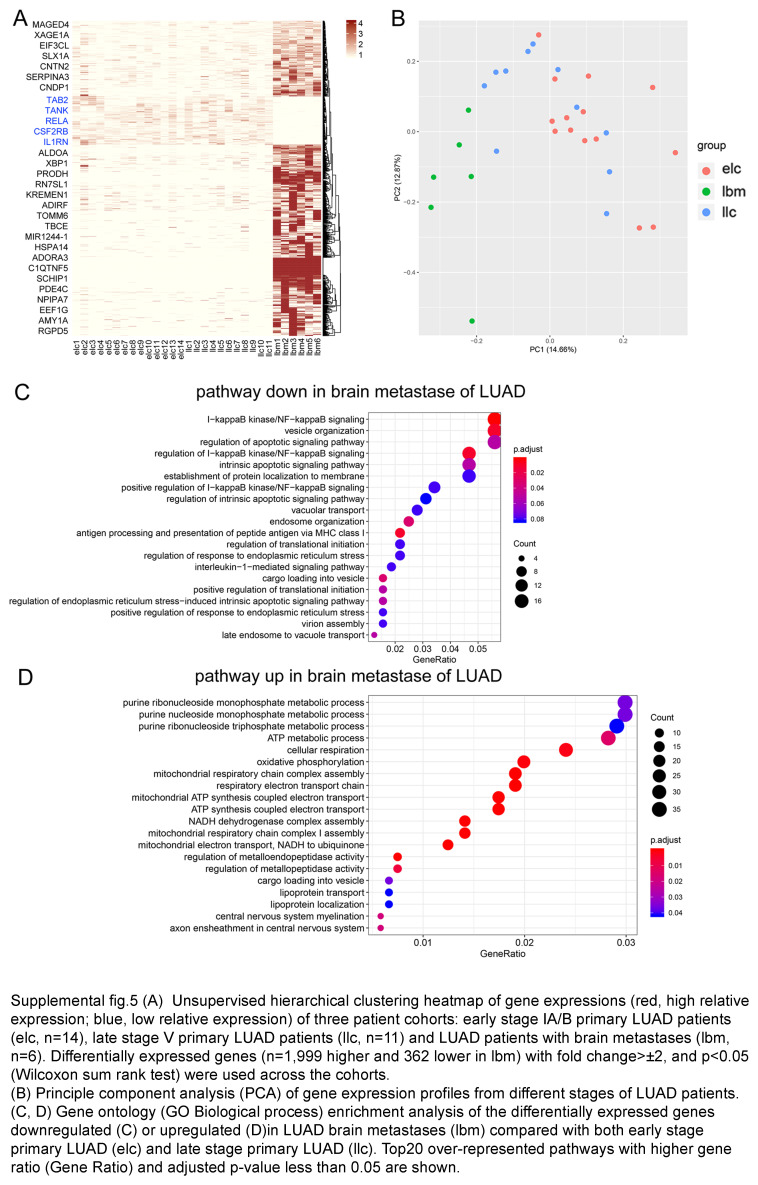


**Figure:**
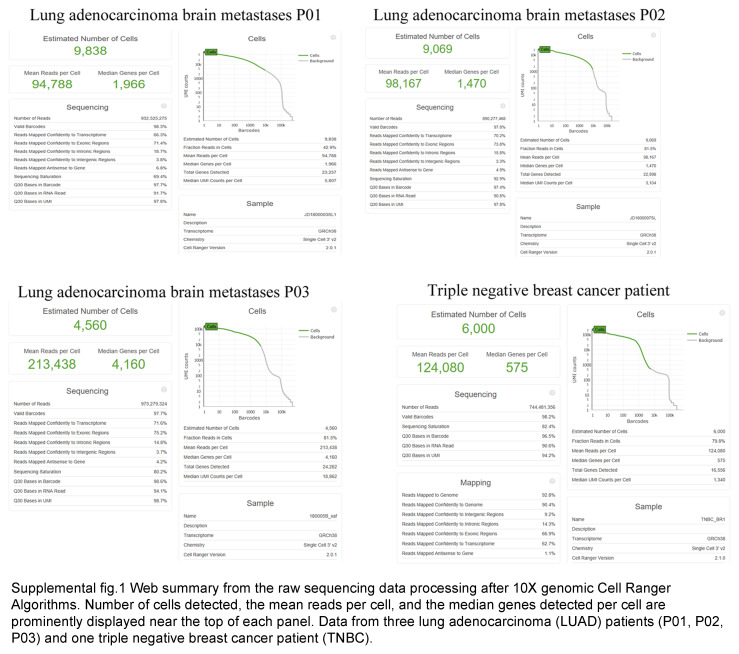


**Figure:**