# Supplementary material for: Single-cell map of diverse immune phenotypes in the metastatic brain tumor microenvironment of nonsmall-cell lung cancer
Source: Int J Surg. 2024 Sep 23;111(1):1601–6. doi: 10.1097/JS9.0000000000002088 (PMC11745726; doi:10.1097/JS9.0000000000002088)
Supplement: Supplementary file 1 [file js9-111-1601-s001.doc]

**Single-Cell Map of Diverse Immune Phenotypes in the Metastatic Brain Tumor Microenvironment of Non-Small-Cell Lung Cancer**

Liang Wang^1, 2#*^, Run-Run Han^3#^, Lei Li ^4,5#^, Lei Dong^6^, Ming-Zhu Jin^7^, Da-yun Feng^1^, Gang Zhu^1^, Wei Guo^1^, Yuan Wang^1^, Min Chao^1^, Fan Chen^1^, Li Gao^1^, Shi-Jia Jin^8^, Dong-Ping Wei ^9^, Wei Sun^10^, Jin-xiang Dai^11*^, Wei-Lin Jin^12,13 *^

1. Department of Neurosurgery, Tangdu Hospital, Fourth Military Medical University, Xi’an 710038, China
2. Frontier Medical Innovation Center，Tangdu Hospital, Fourth Military Medical University, Xi’an 710038, China
3. Shanghai Pulmonary Hospital, Tongji University School of Medicine, Shanghai 200433, China
4. School of Public Health, Health Science Center of Xi'an Jiaotong University, Xi'an, China.
5. Key Laboratory of Trace Elements and Endemic Diseases of National Health Commission and Collaborative Innovation Center of Endemic Diseases and Health Promotion in Silk Road Region, Xi'an, China.
6. Department of Pathology, Ruijin Hospital, Shanghai Jiao Tong University School of Medicine, Shanghai 200025, China
7. Department of Gynecology and Obstetrics, Xinhua Hospital Affiliated to Shanghai Jiao Tong University School of Medicine, Shanghai 200092, China
8. Shanghai Jiao Tong University School of Medicine, Shanghai 200025, China
9. Department of Oncology, Nanjing First Hospital, Nanjing Medical University, Nanjing 210006, China
10. Department of Neurosurgery, Shanghai Institute of Neurosurgery, Changzheng Hospital, Second Military Medical University, Shanghai 200003, China
11. Human Biology Division, Laboratory for the Study of Metastatic Microenvironments, Fred Hutchinson Cancer Research Center, Seattle, USA
12. Key Laboratory for Thin Film and Microfabrication Technology of Ministry of Education, School of Electronic Information and Electronic Engineering, Shanghai Jiao Tong University, Shanghai 200240, China
13. Institute of Cancer Neuroscience, Medical Frontier Innovation Research Center, The First Hospital of Lanzhou University, The First Clinical Medical College of Lanzhou University, Lanzhou 730000, China

# # These authors contributed equally to this study

**Supplemental Methods**

**Human Specimens**

Tumor tissues were obtained from patients undergoing brain resection surgery at our hospital after obtaining informed consent.

**Patient Clinical Characteristics**

Samples were collected from 9 patients who were diagnosed with lung cancer and brain lesions were confirmed as LUAD subtype and 1 TNBC patient with brain metastasis (Supplemental table 2). The 9 LUAD patients consisted of 3 males and 6 females and ranged from age 28 to 62 years old, with a median of 52 years old. The TNBC patient was 44 years old.

**Sample Collection and digestion for single cell RNAseq**

Brain tissue containing the metastatic lesions was collected under surgeries at our Hospital with informed consent under a protocol approved by our hospital Review Board. Tissue samples were placed in DMEM/RPMI media (Corning) on ice. Small chunk of the tissue was processed for immunohistochemistry analysis by quick frozen section. H&E slides were reviewed by a pathologist and confirmed as LUAD brain metastasis or TNBC brain metastasis. The remaining freshly dissected brain tissue was cut up and then enzymatically dissociated to obtain a suspension of cells using a Tumor Dissociation Kit (MiltenyiBiotec#130-095-929) by incubating at 37 ℃ for 15 minutes. Dissociated single cells were pelleted by centrifuged at 130g for 10 minutes after removing unlysed tissue fragments with a 20 μm cell strainer. The cell pellet was then resuspended in Dulbecco’s phosphate-buffered saline (DPBS) containing 0.02% BSA. Cell health was assessed by trypan blue exclusion. Ensure that no less than 90% of the cell samples are alive for subsequent scRNAseq (10X Genomics).

**Single cell capture, library preparation, and sequencing**

Briefly, single cells were partitioned into Gel beads in Emulsion in the 10X Chromium Controller instrument followed by cell lysis and barcoded reverse transcription of RNA, amplification, shearing and 5′ adapter and sample index attachment. On average, ~10,000 cells were loaded on each channel that resulted in the recovery of ~7822 cells. Libraries were sequenced on Illumina HiSeq2500 instruments using paired-end sequencing (PE1 54 bp and PE2 66 bp). Each replicate was sequenced on one half of a HiSeq lane, at an initial depth of approximately 100 million reads.

**RNA isolation and bulk RNAseq**

In brief, total RNA was extracted using Qiagen Total RNA Isolation Kit, and the integrity and concentration were evaluated using an Agilent 2100 Bioanalyzer (Agilent Technologies, USA). Total RNA was purified to obtain mRNA using oligo (dT) magnetic beads. The cDNA libraries were constructed using Illumina TruSeq RNA Sample Prep Kit (Illumina, USA) according to the manufacturer’s protocol. The quality of the constructed cDNA libraries was examined on an Agilent Technologies 2100 382 Bioanalyzer prior to sequencing with Illumina HiSeq™ 2500 at Shanghai OE Biotechnology Co., Ltd. (http://www.oebiotech.com/). Bcl to fastq conversion was done with Illumina bcl2fastq software and demultiplexed into individual files of each sample with self-written scripts in Perl according to the indices for pooling. Low-quality reads were filtered out with Trimmomatic software with default parameters and then clean reads were aligned to the indexed human GRCh38 genome reference with HISAT2 software to generate the SAM files, which were finally converted into sorted BAM files with SAMtools software. Alignment of reads into target genes was performed by using the Stringtie software [ Pertea, M., et al., Transcript-level expression analysis of RNA-seq experiments with HISAT, StringTie and Ballgown. Nat Protoc, 2016. 11(9): p. 1650-67], and gene counts were calculated using the Stringtie/prepDE.py with default options. Log10-transformed TPM (transcript per million reads) was used to calculate and compare gene expression differences between different samples. In this study, p < 0.05 and a fold change (FC) > 2 (log2FC>1) in at least one treatment were set as thresholds to define the significance of gene expression differences between two treatments (Supplementary fig.5A). The Blast2GO software was applied to get the annotation results of unigenes in the Gene Ontology (GO) database. The pathways were also annotated according to the KEGG database.

**Immunohistochemical analysis**

Lung adenocarcinoma with lung tissue microarray, containing 48 cases adenocarcinoma and matched cancer adjacent or adjacent normal lung tissue was purchased from Alenobio (Cat#LC10013b) and brain lesions from 15 patients with LUAD were surgical resected and formalin fixed and paraffin embedded. Immunohistochemistry using standard immunoperoxidase staining was performed on formalin-fixed paraffin-embedded tissue sections (5 μm thick) from specimens of each of the tumor resections. Briefly, we used 3 × 3 min cycles of de-paraffinization in xylene, 2 × 1 min cycles of dehydration in 100% ethanol, 2 × 1 min cycles of dehydration in 95% ethanol, and a 1-min cycle of dehydration in 70% ethanol. Slides were then washed in water. We used 0.01 M citrate buffer (pH 6) for antigen retrieval in a microwaved pressure cooker for 20 min. We then washed the slides three times in phosphate-buffered saline (PBS) after cooling for 30 min. We quenched endogenous peroxidase in 3% hydrogen peroxide in PBS for 10 min, washed three times in PBS, and blocked with 10% goat serum for 25 min. We then incubated the slides with primary antibodies for 90 min at room temperature. We used the following primary antibodies: mouse anti-CD20 (ORIGENE, ZM-0039, 1:100 dilution), anti-CD3 (ORIGENE, ZM-0417,1:100 dilution), anti-CD68 (ORIGENE, ZM-0464, 1:100 dilution). After washing three times in PBS, we incubated the slides with biotinylated goat anti-rabbit secondary antibody (Vector Laboratories, 1:200 dilution) for 30 min at room temperature, followed by additional PBS washing, 30-min incubation with ABC peroxidase reagent, development in DAB-peroxidase substrate solution (DAKO), and counter-staining in hematoxylin. Images were taken at 10X5 by LEICA DMC 4500 and analyzed using Image-Pro Plus 6.0.

**Supplemental figure legend**

**Supplemental fig.1**

Web summary from the raw sequencing data processing after 10X genomic Cell Ranger [Algorithms](https://support.10xgenomics.com/single-cell-gene-expression/software/pipelines/latest/algorithms/overview#cell_calling). Number of cells detected, the mean reads per cell, and the median genes detected per cell are prominently displayed near the top of each panel. Data from three lung adenocarcinoma (LUAD) patients (P01, P02, P03) and one triple negative breast cancer patient (TNBC).

**Supplemental fig.2**

(A) Relative leukocyte fractions evaluated by CIBERSORT in our bulk RNA-seq data to infer relative RNA fractions from 22 leukocyte subsets (LM22 signature) in each patient represented with different colors.

(B) tSNE plot color-coded for expression (low to high: gray to dark red) of marker genes. Clusters are indicated in figure 1B. Cytotoxic T cells:CD3E, GMZB and ENTPDA(CD39); Macrophages: CD68, CSFR1, CD14, AIF1, CCL3, CCL4, CXCL9 and CXCL10.

**Supplemental fig.3**

Immunohistochemistry assessment of formalin-fixed paraffin embedded archival tissue samples (×200 magnification). For each patient, samples were stained with CD3 (T cell marker), CD20 (B cell marker) and CD68 (macrophage marker), respectively. Representative images shown as (A) six early stage primary LUADs (elc, stage IA/B); (B) six late stage primary LUADs (llc, stage IV) and six brain metastatic lesions from treatment naïve LUAD patients (lbm). Scale bar:200um.

**Supplemental fig.4**

Gene ontology (biological process) analysis of the differentially expressed genes enriched in TAM clusters in different stages of LUAD:(A) Early stage primary LUAD (elc, cluster 9); (B) Late stage primary LUAD (llc, cluster 5); (C) LUAD brain metastases (lbm, cluster 20). Top20 over-represented GO terms with higher enrichment scores and FDR (false discovery rate) significance less than 0.05are shown.

**Supplemental fig.5**

# (A)  Unsupervised hierarchical clustering heatmap of gene expressions (red, high relative expression; blue, low relative expression) of three patient cohorts: early stage IA/B primary LUAD patients (elc, n=14), late stage V primary LUAD patients (llc, n=11) and LUAD patients with brain metastases (lbm, n=6). Differentially expressed genes (n=1,999 higher and 362 lower in lbm) with fold change>±2, and p<0.05 (Wilcoxon sum rank test) were used across the cohorts.

# (B) Principle component analysis (PCA) of gene expression profiles from different stages of LUAD patients.

(C, D) Gene ontology (GO Biological process) enrichment analysis of the differentially expressed genes downregulated (C) or upregulated (D)in LUAD brain metastases (lbm) compared with both early stage primary LUAD (elc) and late stage primary LUAD (llc). Top20 over-represented pathways with higher gene ratio (Gene Ratio) and adjusted p-value less than 0.05 are shown.

**Supplemental fig.6**

(A) Heatmap showing the clustering based on the frequency of V gene (left panel) and J gene (right panel).

(B) Frequency of V gene usage for TCRβ clones across three different patients.

(C) Analysis of TCR clone diversity based on TCR VDJ rearrangement. The number of VDJ gene combinations are counted in three different patients (top panel). The pie graph shows the frequency of representative VDJ combinations that account for more than 1% of total.

(D) Analysis of TCR clone diversity based on specific CDR3 sequence variety. The number of specific CDR3 sequences were counted in three different patients (top panel). The pie graph shows the frequency of specific CDR3 sequences that account for more than 1% of total.
